# Using mice to model Alzheimer's dementia: an overview of the clinical disease and the preclinical behavioral changes in 10 mouse models

**DOI:** 10.3389/fgene.2014.00088

**Published:** 2014-04-23

**Authors:** Scott J. Webster, Adam D. Bachstetter, Peter T. Nelson, Frederick A. Schmitt, Linda J. Van Eldik

**Affiliations:** ^1^Sanders-Brown Center on Aging, University of KentuckyLexington, KY, USA; ^2^Division of Neuropathology, Department of Pathology and Laboratory Medicine, University of KentuckyLexington, KY, USA; ^3^Department of Neurology, University of KentuckyLexington, KY, USA; ^4^Department of Anatomy and Neurobiology, University of KentuckyLexington, KY, USA

**Keywords:** Alzheimer's disease, mouse models, neuropsychological assessment, behavior, cognition, APP mice, APP/PS1 mice, 3×TG-AD mice

## Abstract

The goal of this review is to discuss how behavioral tests in mice relate to the pathological and neuropsychological features seen in human Alzheimer's disease (AD), and present a comprehensive analysis of the temporal progression of behavioral impairments in commonly used AD mouse models that contain mutations in amyloid precursor protein (APP). We begin with a brief overview of the neuropathological changes seen in the AD brain and an outline of some of the clinical neuropsychological assessments used to measure cognitive deficits associated with the disease. This is followed by a critical assessment of behavioral tasks that are used in AD mice to model the cognitive changes seen in the human disease. Behavioral tests discussed include spatial memory tests [Morris water maze (MWM), radial arm water maze (RAWM), Barnes maze], associative learning tasks (passive avoidance, fear conditioning), alternation tasks (Y-Maze/T-Maze), recognition memory tasks (Novel Object Recognition), attentional tasks (3 and 5 choice serial reaction time), set-shifting tasks, and reversal learning tasks. We discuss the strengths and weaknesses of each of these behavioral tasks, and how they may correlate with clinical assessments in humans. Finally, the temporal progression of both cognitive and non-cognitive deficits in 10 AD mouse models (PDAPP, TG2576, APP23, TgCRND8, J20, APP/PS1, TG2576 + PS1 (M146L), APP/PS1 KI, 5×FAD, and 3×Tg-AD) are discussed in detail. Mouse models of AD and the behavioral tasks used in conjunction with those models are immensely important in contributing to our knowledge of disease progression and are a useful tool to study AD pathophysiology and the resulting cognitive deficits. However, investigators need to be aware of the potential weaknesses of the available preclinical models in terms of their ability to model cognitive changes observed in human AD. It is our hope that this review will assist investigators in selecting an appropriate mouse model, and accompanying behavioral paradigms to investigate different aspects of AD pathology and disease progression.

## Introduction

Alzheimer's disease (AD) is characterized by a progressive decline in cognitive function, usually starting with memory complaints and eventually progressing to involve multiple cognitive, neuropsychological and behavioral domains. The definitive diagnosis of AD comes from postmortem analysis of the neuropathological changes in the brain. Analyses of both clinical and pathological features, i.e., clinicopathological correlation studies, have provided important insights into how the pathology correlates with cognitive status. Complementing these studies in humans has been the development of preclinical model systems of AD pathology. These preclinical animal models, especially mouse models, have been extremely useful to test mechanistic hypotheses about AD pathophysiology and to predict outcomes from pharmacological interventions. However, no animal model recapitulates the entirety of AD in humans, and therefore it is important to understand both the utility and limitations of particular animal models. With this in mind, we will present an overview of the neuropathological changes seen in the AD patient population as individuals transition from normal cognitive aging to dementia, review the clinical neuropsychological assessments used in the AD field, review the mouse behavioral tasks commonly used in preclinical testing and discuss how they relate to these clinical neuropsychological assessments, and outline the temporal progression of cognitive and non-cognitive deficits seen in the commonly used mouse models of AD.

## Overview of neuropathological changes in AD

In 2012, new consensus guidelines for neuropathologic evaluation of AD were adopted (Hyman et al., 2012; Montine et al., 2012). The AD neuropathologic change is now ranked on three parameters (Amyloid, Braak, CERAD) to obtain an “ABC” score: histopathologic assessments of beta-amyloid (Aβ)-containing amyloid plaques (A), Braak staging of neurofibrillary tangles (B), and scoring of neuritic amyloid plaques (C). Standard approaches for the workup of cases, preferred staining methods, reporting of results and clinicopathological correlations are also recommended. Unlike the prior AD neuropathologic criteria (Hyman and Trojanowski, 1997) that required a history of dementia, the current guidelines recognize that AD neuropathologic changes can be present in the brain in the apparent absence of cognitive impairment. The updated guidelines thus emphasize the continuum of neuropathologic changes that underlie AD. For a disease process that is known to occur over a decade or more (Blennow and Zetterberg, 2013; Rosen and Zetterberg, 2013; Rosen et al., 2013), and encompasses the age range where people are likely to die of other causes, it is inevitable that many people will die in a prodromal or “preclinical” stage of AD. This consideration points to the complexity that clinicopathological studies face when examining AD pathological contributions to cognitive deficits (Nelson et al., 2009b).

There have been numerous clinicopathological studies attempting to correlate amyloid plaques with the cognitive deficits seen in AD (Blessed et al., 1968; Tomlinson et al., 1970; Duyckaerts et al., 1990, 1998; Berg et al., 1998; Gold et al., 2000; Mungas et al., 2001; Tiraboschi et al., 2002, 2004; Guillozet et al., 2003; Kraybill et al., 2005; Holtzer et al., 2006; Markesbery et al., 2006; Nelson et al., 2007a, 2009a; Beach et al., 2009; Sabbagh et al., 2010; Robinson et al., 2011). Apparent inconsistencies in the conclusions of these studies are due to differences in study cohorts, methodology used to classify plaque subcategories, plaque-counting techniques, and metrics used to assess cognitive deficits. Nevertheless, several important concepts have emerged pertaining to plaque pathology and cognition. First, the strongest correlation between amyloid plaques and cognition is in the early stages of the disease and this association weakens as NFTs and gross neocortical neurodegeneration become more widespread (Thal et al., 2002; Nelson et al., 2009b, 2012). As the disease progresses into the later stages, there is little evidence to support a continued contribution by amyloid plaques to the late-stage AD cognitive decline (Nelson et al., 2009b, 2012). Second, it appears that density of neuritic plaques correlates more strongly with the cognitive deficits than do “diffuse” amyloid plaques (Mckee et al., 1991; Crystal et al., 1993; Tiraboschi et al., 2004; Nelson et al., 2007a; Braak et al., 2011).

In contrast to the literature concerning amyloid plaques, a large number of studies have arrived at a common finding, namely, there is a strong link between neocortical NFTs and cognitive decline (Tomlinson et al., 1970; Duyckaerts et al., 1990, 1997, 1998; Mckee et al., 1991; Arriagada et al., 1992; Bierer et al., 1995; Davis et al., 1995; Dickson et al., 1995; Nagy et al., 1995, 1999; Cummings et al., 1996; Berg et al., 1998; Grober et al., 1999; Sabbagh et al., 1999; Gold et al., 2000; Mungas et al., 2001; Riley et al., 2002; Silver et al., 2002; Tiraboschi et al., 2002; Guillozet et al., 2003; Bennett et al., 2004; Kraybill et al., 2005; Holtzer et al., 2006; Markesbery et al., 2006; Koepsell et al., 2008; Whitwell et al., 2008; Beach et al., 2009; Brayne et al., 2009; Giannakopoulos et al., 2009; Sabbagh et al., 2010; Robinson et al., 2011). It should be noted that outside of frontotemporal lobar degeneration (FTLD), one does not see widespread cortical NFTs without abundant plaque pathology. In the earliest stages of AD (Braak stage I-II), NFTs are restricted to the entorhinal cortex (Braak and Del Tredici, 2011; Braak et al., 2011). NFTs spread to the limbic and medial temporal lobe (Braak stage III-IV), and this stage correlates with early AD symptoms related to memory (Schmitt et al., 2000; Riley et al., 2011). During the late stages (Braak stage V–VI), NFTs increase in number and manifest in neocortical areas responsible for higher cognitive domains such as executive function, visuospatial capacities, and speech in synchrony with observed AD-related cognitive deficits in these respective cognitive domains. Not all AD cases fall within the standard NFT distribution described by the Braak staging (Hof et al., 1997; Abner et al., 2011; Murray et al., 2011). Some cases classified as “high level” of AD neuropathological changes may show subtle or undetectable cognitive impairment, yet all cases with quantifiably “end stage” neocortical NFT pathology show cognitive impairment (Dickson et al., 1995; Berlau et al., 2007; Nelson et al., 2009a, 2012; Abner et al., 2011; Santacruz et al., 2011; Jicha et al., 2012). In sum, the correlations noted in human material support the hypothesis that plaques and tangles correlate with cognitive status. The data also support the “Amyloid Cascade Hypothesis”(Karran et al., 2011), a deceptively complex hypothesis which posits that beta-amyloid/plaque pathology kindles widespread tau/NFT pathology, with the tau/NFT pathology constituting the more direct cause of the cell loss and synapse elimination underlying clinical disease (Nelson et al., 2009b, 2012).

## Cognitive neuropsychological assessments used in the AD field

Neuropsychological assessment is the most reliable means to clinically evaluate the cognitive deficits seen in humans. Many neuropsychological tests have been developed which are highly sensitive to the cognitive behavioral symptoms seen in AD, and these tests are extensively used as clinical diagnostic tools (Schmitt, 1994) as well as to track the progression of the disease (Flicker et al., 1984; Morris et al., 1989; Storandt and Hill, 1989; Storandt, 1991; Welsh et al., 1991, 1992; Locascio et al., 1995; Albert, 1996; Storandt et al., 1998; Schmitt et al., 2000; Salmon and Bondi, 2009). Current neuropsychological assessments (from the National Institute on Aging workgroups on diagnostic guidelines for AD) aim to detect disruptions in cognitive domains such as episodic memory, semantic memory, working memory, and attention, as well as dysfunction in language, praxis, and executive functioning (Flicker et al., 1984; Baddeley et al., 1986, 1991, 2001; Huff et al., 1987; Knopman and Ryberg, 1989; Hodges et al., 1992; Parasuraman and Nestor, 1993; Hodges and Patterson, 1995; Perry and Hodges, 1999; Salmon et al., 1999; Perry et al., 2000; Backman et al., 2001; Lambon Ralph et al., 2003). In the following section, we will cover several of the most common neuropsychological tests used clinically to assess the mental status and memory disruptions in AD (for more in depth reviews see Perry and Hodges, 1999; Budson and Price, 2005; Bondi et al., 2008; Weintraub et al., 2012). Table 1 provides an overview of four mental status examinations and two brief memory tests commonly used clinically.

**Table 1:** **Common neuropsychological assessment tasks seen clinically**.

**Task**	**Description**	**Cognitive domains**	**References**
**MENTAL STATUS EXAMS**			
Mini-Mental State Examination (MMSE)	Nineteen item (30 points) test of general cognitive status	Working memory, attention, memory (semantic), praxis, etc.	Folstein et al., 1975
Montreal Cognitive Assessment (MoCA)	A rapid screening method to assess mild cognitive dysfunction	Working memory, memory (semantic and episodic), attention, visuospatial memory, etc.	Nasreddine et al., 2005
Short Blessed Test (SBT)	A short six item test measuring general cognitive status	Memory (semantic and episodic), working memory, and attention, etc.	Blessed et al., 1968
Alzheimer's Disease Assessment Scale (ADAS)	An 11 part test that measures cognitive dysfunction	Memory (semantic and episodic), and attention, etc.	Rosen et al., 1984
**MEMORY TESTS**			
Logical memory test I and II	A short story is presented to the patient and used to test immediate memory (test I) and delayed memory (test II)	Memory (episodic), verbal recall, etc.	Wechsler, 1997
Benton Visual Retention Test (BRVT)	Visual based test of general memory	Memory (episodic), and working memory etc.	Benton, 1992

### Mental status exams

Mental status examinations assess multiple mental functions and cognitive abilities across multiple domains (see Table 1), and are generally more encompassing than specific verbal and visual memory tests (examples also seen in Table 1). Both categories of tests are clinically useful in assessing the cognitive progression of AD. Most mental status examinations assess mental functions and cognitive ability across a wide range of areas such as: language skills, arithmetic ability, visuospatial ability, attention, memory, and orientation to time and place.

The Mini-Mental Status Examination (MMSE) is one of the most commonly used neuropsychological screening tools for cognitive impairments seen in AD (Simard and Van Reekum, 1999; Snyderman and Rovner, 2009). The MMSE is a brief questionnaire that can both diagnose cognitive impairment and track the severity of this cognitive impairment throughout the pathogenesis of the disease. The MMSE covers multiple areas such as: attention, memory (semantic and episodic), orientation to time and place, and working memory. The scoring system ranges from 0 to 30 points. In general, a score of 27 or greater reflects normal cognition, a score of 19–24 represents mild impairment, a score of 10–18 represents moderate impairment, and a score below 9 indicates severe cognitive impairment. The MMSE has excellent reproducibility that lends itself well to longitudinal use in tracking the progression of the cognitive impairments associated with AD (Jacqmin-Gadda et al., 1997; Aevarsson and Skoog, 2000; Chatfield et al., 2007).

The Montreal Cognitive Assessment (MoCA) is a more recent mental status examination used for cognitive dysfunction seen in AD. This test battery takes approximately 10 min to administer and covers many similar cognitive domains to that of the MMSE. The total possible score is 30 points, with a score of 26 or above considered within the normal range. An example of a task included in the MoCA is the forward and backward digit span test. In the forward digit span test, a sequence of five numbers is read at a rate of one number per second and the test taker is required to repeat a set of numbers in exactly the same sequence as they were presented. In the backward digit span task, the test taker is required to repeat a three number sequence in the reverse order in which it was presented. One point is awarded for each of the digit span tests in which the test taker made no errors. The MoCA also includes a delayed recall memory test. Relatively near the beginning of the test, the examiner presents a short word list for the patient to remember. At the end of the test, the patient is again prompted to recall the word list and for each of the words correctly remembered one point is awarded.

Another commonly used test battery is that of the Short Blessed Test (SBT) which consists of a six-item test designed to identify the cognitive dysfunction seen in AD (Katzman et al., 1983). An appealing advantage of the SBT is the ease and speed with which it can be administered (often taking only a few minutes). In the SBT, errors are scored for incorrect answers and the scoring range falls between 0 and 28, with a score of 0–4 representing normal cognition, a score of 5–9 representing early impairment, and a score of 10 or more representing impaired cognition. Despite the simplicity and brevity of the SBT, the results that it produces have demonstrated excellent reliability (Fuld, 1978). Similarly, the SBT was the first mental status examination to be correlated with amyloid plaque burden at autopsy (Carpenter et al., 2011).

The Alzheimer's Disease Assessment Scale (ADAS) was specifically developed to measure the severity of symptoms commonly seen in AD (Rosen et al., 1984). Initially, it was developed in two parts (sub-scales): one for cognitive symptoms and one for non-cognitive symptoms. The cognitive sub-scale of the ADAS is commonly referred to as the ADAS-cog, and has become one of the most common neuropsychological tests used to assess AD progression. The scoring for the ADAS-cog ranges from 0 to 70, with a low score representing a cognitively intact person and a high score representing someone with cognitive impairment. Because the ADAS-cog has an excellent test-retest reliability and is considered to be one of the most sensitive scales for assessing cognitive changes related to AD (Emilien et al., 2004), this test is extensively used in AD clinical trials as an outcome measure of cognitive change (Schmitt and Wichems, 2006; Connor and Sabbagh, 2008).

### Memory tests

Individual memory tests are generally shorter than mental status examinations and focus solely on assessing memory deficits. Both verbal and visual memory tests are commonly used clinically as stand alone tests or incorporated into a more comprehensive mental status examination. Examples of such memory tests are the Logical Memory Test I and II (LM-I and LM-II) and the Benton Visual Retention Test (BVRT) (Benton, 1992; Wechsler, 1997).

The LM-I and LM-II were originally developed as subtests to the Wechsler memory scale, but are commonly used as stand-alone memory tests. Both are verbal based memory tests that involve a short story read to the patient. In the LM-I, the patient answers immediate questions related to the narrative, whereas in the LM-II there is a delay between the presentation of the story and the questions. Thus, these memory tests are used to assess immediate recall (LM-I) and delayed recall (LM-II).

In the BVRT, the patient is shown 10 different visual designs, one at a time, and is then asked to reproduce each one from memory exactly as it appeared. While scoring the BVRT, errors of omissions, distortions, perseverations, rotations, misplacements, and size are all looked for and can give some insight into the progression of the disease. For example, if the patient has a high number of perseveration errors it is likely that the AD pathology has manifest itself in neocortical areas responsible for higher cognitive domains such as executive function, and visuospatial capacities (Braak Stage V–VI).

### Cognitive neuropsychological assessment summary

**Figure 1:**
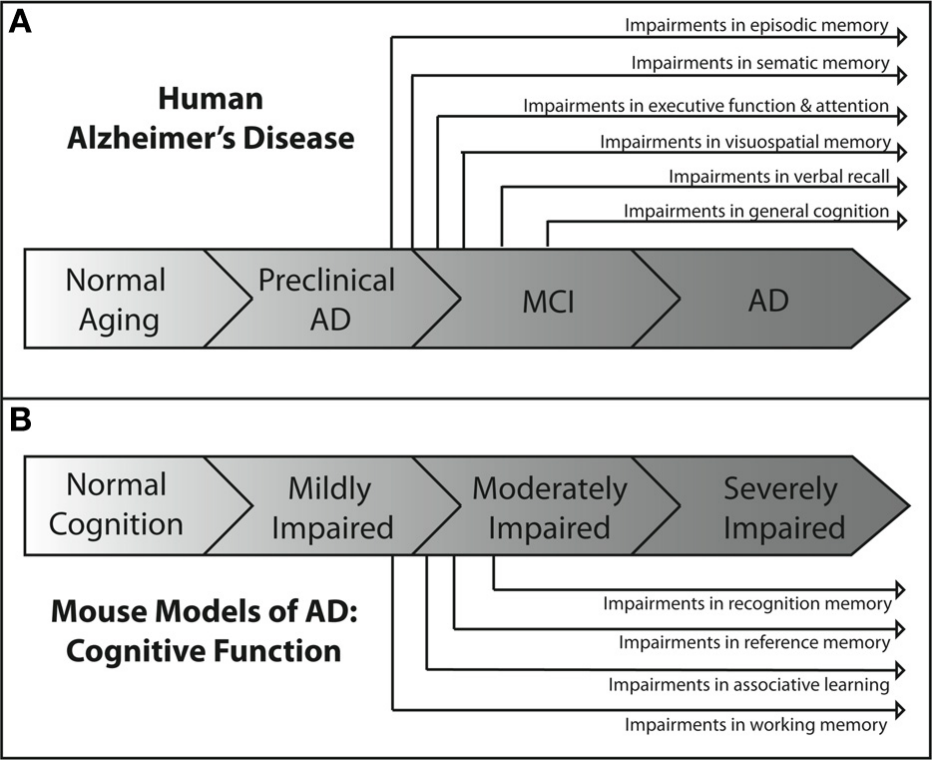
**Overview of the progression of cognitive deficits in human AD and in mouse models of AD. (A)** In the human disease, the earliest AD-related cognitive deficits present themselves as episodic memory impairment during the late preclinical phase of the disease (Backman et al., 2005; Twamley et al., 2006; Bondi et al., 2008). Semantic memory deficits are the next to develop (Tuokko et al., 2005; Storandt et al., 2006), followed by impairments in executive functioning (Bondi et al., 2008), attention (Bondi et al., 2008), and visuospatial memory (Twamley et al., 2006) near the beginning of the MCI phase of the disease. As MCI progresses, deficits in verbal recall (Kryscio et al., 2006; Bondi et al., 2008) develop and impairments in general cognition (Bondi et al., 2008) become apparent. As the patient transitions into AD, all cognitive domains become affected. **(B)** The development of cognitive deficits in APP mouse models of AD shows similar patterns of progression. Consistently, the earliest observable impairments are in spatial working memory (Webster et al., 2013), as assessed through the use of water maze based tasks. These impairments are generally followed temporally by impairments in associative learning and reference memory, as assessed by maze alternation (Lalonde et al., 2012) and fear conditioning tasks (Kobayashi and Chen, 2005). Deficits in recognition memory usually present later in the spectrum of cognitive impairment than deficits in other domains (Eriksen and Janus, 2007; Hall and Roberson, 2012; Webster et al., 2013).

Each of the tests described above is aimed at assessing deficits in different cognitive domains. Each of these domains has been shown to be impaired at some point in the spectrum of human AD. However, they are not uniformly affected throughout the course of the disease. Deficits in some domains occur relatively early, while deficits in others occur much later in the progression of the disease. Figure 1A depicts an overview of the time course of affected cognitive domains in human AD. It has become increasingly clear that identifying and targeting the cognitive deficits that occur early in the course of the disease is critical to producing the maximum impact of treatment on cognitive function and quality of life (Salmon et al., 2002). Thus, great efforts have been made to better understand the profile of cognitive deficits associated with early AD, and have resulted in earlier and more reliable clinical diagnosis (Bondi et al., 1995, 1999; Jacobson et al., 2002; Lange et al., 2002; Mickes et al., 2007).

Some of the earliest neuropathological changes in AD are in the hippocampus and entorhinal cortex, followed by changes in the medial temporal lobe. Consistent with this progression of pathology, the earliest detectable deficits in cognition are seen in medial temporal lobe-dependent episodic memory (Bondi et al., 1999; Collie and Maruff, 2000; Schmitt et al., 2000; Smith et al., 2007). These early deficits in episodic memory are followed closely by deficits in semantic memory, and both are developed before other deficits in cognitive domains such as attention, visuospatial memory, or executive function (Bondi et al., 2008). This suggests that cognitive functions such as episodic and semantic memory that depend heavily on the neural circuitry of the medial and lateral temporal lobes may be impaired earlier than cognitive abilities that depend on the circuitry of other brain regions. Further support for this idea comes from the time course of the frontal lobe dependent executive function deficits observed in patients. Slight deficits in executive functioning are first detectable near the end of the preclinical phase of AD but after the observed deficits in episodic and semantic memory (Storandt et al., 2006; Twamley et al., 2006). As the patient moves from the preclinical phase of AD into MCI, more cognitive domains begin to be affected. Most studies of MCI patients show consistent impairments in verbal recall (Larrieu et al., 2002; Tuokko et al., 2005; Kryscio et al., 2006) and a decline in general memory functioning (Tuokko et al., 2005; Bondi et al., 2008). Once the AD patient progresses past MCI and into dementia, general cognition continues to decline with deficits appearing in all respective cognitive domains (Huff et al., 1987; Locascio et al., 1995; Lambon Ralph et al., 2003; Mckhann et al., 2011).

The importance of neuropsychological testing cannot be overstated, as it is the only measure that provides information about a patient's current cognitive status and remains the most reliable means to clinically diagnose probable AD. Neuropsychological testing provides information on both general cognitive status and specific information on different cognitive domains affected in AD. Composite scores encompassing multiple neuropsychological tests are often used and can provide some of the most reliable assessments of global cognitive status relating to AD as well as serving as efficacy outcome measures in clinical trials (Bernick et al., 2012).

## How commonly used preclinical mouse behavioral tasks relate to the clinical neuropsychological assessment tests in human AD?

Ideally, preclinical rodent cognitive testing would assess identical cognitive domains to those examined through neuropsychological testing in human AD. Indeed, many rodent behavioral tasks have been specifically designed with this in mind, and while each task varies with respect to face, construct, and predictive validity, they all attempt to model different aspects of the cognition disrupted in AD and targeted by the human neuropsychological assessments listed in the previous section. Some cognitive domains disrupted in AD have been extensively modeled (reference memory, working memory and executive function), some less so (attention), and some nearly not at all (episodic memory). Reference memory, while not used clinically to describe human cognition, refers to learned knowledge for an aspect of a task that remains constant throughout the behavioral task and most closely correlates to human semantic memory. Working memory refers to a mental processing system used to hold transitory information for a limited time where it can be manipulated and operated on and used to guide behavior. Recognition memory refers to the ability to recognize previously encountered events, objects, or individuals and is classified as part of long-term declarative memory. Other cognitive domains impaired in human AD such as those involving language (i.e., verbal acuity tasks and verbal recall tasks) simply cannot be modeled in rodent models. Table 2 provides a short description of various behavioral tasks used to assess AD-like cognitive deficits in mice, and summarizes the respective cognitive domains measured by each task.

**Table 2:** **Commonly used mouse behavioral tasks**.

**Task**	**Description**	**Cognitive domains**	**References**
Morris Water Maze (MWM)	Widely used behavioral task where mice are placed in a circular pool and must find a hidden escape platform	Reference memory and working memory	Morris et al., 1982
Radial Arm Maze (RAM)	The maze usually consists of 6–8 arms radiating from a round central space. Various arms are baited with a food reward.	Reference memory and working memory	Olton and Samuelson, 1976
Radial Arm Water Maze (RAWM)	A submerged version of the RAM where the food reward is replaced by an escape platform.	Reference memory and working memory	Diamond et al., 1999
Barnes maze	Consists of a circular platform with holes around the circumference and an escape box	Reference memory and working memory	Barnes, 1979
T-Maze/Y-Maze alternation	A three arm maze which forces the animal to choose between two arms	Reference memory and working memory	Blodgett and Mccutchan, 1947; Glickman and Jensen, 1961
Novel Object Recognition (NOR)	A two trial memory task which uses the animal's innate exploratory behavior to assess memory	Recognition memory	Ennaceur and Delacour, 1988
Contextual and cued fear conditioning	The animal learns to predict an aversive stimulus based on an associated context/cue	Reference memory (associative learning/memory)	Fanselow, 1980; Curzon et al., 2009
Passive avoidance	An avoidance task where the animal must refrain from entering a chamber where an aversive stimulus was previously administered	Reference memory (associative learning/memory)	Van Der Poel, 1967
Active avoidance	A fear-motivated associative avoidance test where an animal must actively avoid an aversive stimulus	Reference memory and working memory (associative learning/memory)	Vanderwolf, 1964
Delayed Matching (non-matching) to Position/Sample (DMTP/DMTS)	The animal receives a sample stimulus and then after a short delay is required to choose the correct corresponding response	Working memory	Dunnett, 1993; Robinson and Crawley, 1993
Multiple-Choice Serial Reaction Time Task (CSRTT)	The animal must attend to several spatial locations (usually 3–5), observe a corresponding stimulus, and then correctly respond	Attention, impulsivity, and executive function	Carli et al., 1983
Attentional set-shifting tasks	The animal must shift back and forth between changing rules to successfully obtain a reward	Executive function and cognitive flexibility	Birrell and Brown, 2000
Reversal learning	Adjustment to changes in reward contingency	Executive function and working memory	Butter, 1969; Bussey et al., 1997
What-Where-Which Task (WWWhich)	The animal must associate an object (What) with its location (Where) in a specific visuospatial context (Which) to form an integrated memory	Recognition memory and episodic-like memory	Davis et al., 2013a,b

### Modeling working memory

Working memory is perhaps one of the most well modeled aspects of the memory deficits in AD. Clinically, many of the neuropsychological tests that assess working memory rely heavily on the use of verbal tasks (Kaplan et al., 1978; Benedict et al., 1998; Spreen and Strauss, 1998; Delis et al., 2000), employing language as a core construct and thus are not feasible to model using mice. Instead, spatial based working memory tasks are heavily employed in murine working memory testing and likely are more depictive of the visuospatial working memory tasks used clinically (Benton, 1992; Benedict and Groninger, 1994).

The most widely used paradigms for working memory in mice are maze type tasks which require spatial working memory to solve. The earliest variants of these are the T-maze and Y-maze alternation tasks, which are relatively simple tests consisting of three arms with a single intersection. These tasks rely on the natural exploratory behavior (tendency to choose an alternative arm over an arm which has been previously explored) of rodents and are considered the most rudimentary tasks to assess spatial working memory (Dudchenko, 2004). A more complex maze type task used to test murine spatial working memory is that of the Radial Arm Maze (RAM), consisting of several arms (usually 6–8) radiating outwards from a central platform (Olton and Samuelson, 1976; Olton et al., 1979). In the RAM, the animal is started in the center area and then some of the arms or all of the arms can be baited with a food reward. Depending on the baiting paradigm, unimpaired rats and mice will quickly learn where the food reward is and which arms have previously been visited, and will avoid re-entering a previously entered arm. Perhaps the most widely employed spatial working memory task is that of the Morris Water Maze (MWM) (Morris et al., 1982). The MWM consists of a large open pool with a hidden (submerged) escape platform located somewhere within the pool. Animals must learn where the platform is, remember the platform's location, and then use spatial cues on subsequent trials to navigate back to the hidden platform. Large numbers of AD mouse models have been tested in the MWM and most show AD related cognitive deficits (Webster et al., 2013). Other common tasks of murine spatial working memory are the Radial Arm Water Maze (RAWM) and the Barnes Maze (Barnes, 1979; Diamond et al., 1999; Alamed et al., 2006).

It is important to note that while many of the previously described behavioral tasks are considered tasks of working memory they can also be modified to test reference memory depending on the testing protocol used. Similarly, not all models of murine working memory are spatial working memory tasks. For example, there exist versions of both the RAM and RAWM that are considered non-spatial working memory tasks (Olton and Feustle, 1981; Crusio et al., 1993; Hyde et al., 2000). Other examples of non-spatial working memory tasks are operant tasks such as the Delayed Match to Sample (DMTS), Delayed Non-Match to Sample (DNMTS), and Delayed Stimulus Discrimination Task (DSDT) (Dudchenko, 2004; Buccafusco et al., 2008). In these non-spatial working memory tasks the animal is required to remember a stimulus (over a delay period) that is paired with a particular type of response (generally a lever press or a nose poke) and a correct response is rewarded. In many of the non-spatial operant working memory tasks each animal can perform many trials per day and thus can serve as its own control. This lends itself nicely to pharmacological based studies assessing potential therapeutic compounds for the treatment of the cognitive deficits seen in AD (Buccafusco et al., 2008).

### Modeling executive function

Executive function refers to a broad range of higher cognitive processes such as: reasoning, planning, cognitive flexibility, sequencing, response inhibition, and abstract concept formation. The current mouse models of executive function most closely replicate the human aspects of cognitive flexibility and response inhibition in executive function. Attentional set-shifting tasks are one of the main behavioral tasks used to assess executive function in the mouse. In many ways, set-shifting tasks are similar to the Wisconsin Card Sorting Task in that they form the gold standard for assessing executive function (Drewe, 1974; Robinson et al., 1980; Arnett et al., 1994). In both the Wisconsin Card Sorting Task in humans and set-shifting tasks in mice, the dorsolateral and orbital prefrontal cortex is critical for successful performance (Weinberger et al., 1986; Berman et al., 1995; Brigman et al., 2005; Bissonette et al., 2008). In the most common version of the murine set-shifting task, mice are required to select a bowl in which to dig for a food reward. Bowls can be discriminated from each other according to different stimulus dimensions such as texture and odor. Successful completion of the task requires the animal to shift between stimuli dimensions to successfully retrieve the food reward. The ability to extract knowledge from different stimuli dimensions suggests that the mouse is capable of using at least some aspects of higher-order cognitive functions seen in human executive functioning (Chudasama, 2011). Numerous different transgenic mouse models of AD have shown deficits in set-shifting tasks (Zhuo et al., 2007, 2008; Marchese et al., 2013).

Reversal learning is another way that aspects of executive function are modeled in the mouse. While less complex than attentional set shifting, reversal learning does require both cognitive flexibility and impulse control, thus tapping into components of human executive function (Chudasama, 2011; Stopford et al., 2012). There are many different variations of reversal learning tasks in mouse behavior, but they all work on the same principle. The animal first learns that a particular response to a stimulus will be rewarded, while a response to a different stimulus will be unrewarded. Then the stimulus-reward is switched so that the previously unrewarded stimulus becomes the rewarded stimulus. The animal must learn to reverse responses in order to receive the reward. Wild type control mice are able to quickly adjust their response in order to obtain the reward. However, animals with prefrontal cortex lesions display profound deficits in reversal learning (Chudasama, 2011; Izquierdo and Jentsch, 2012). Similarly, many different AD mouse models have shown impairment in reversal learning (Angelo et al., 2003; Dong et al., 2005; Filali et al., 2012; Cheng et al., 2013; Musilli et al., 2013; Papadopoulos et al., 2013).

Another aspect of executive function that is modeled in mice is response inhibition. Response inhibition is required for the appropriate control of an individual's behavioral actions in response to a stimulus (Robbins, 1996; Humby et al., 1999; Perry and Hodges, 1999; Romberg et al., 2013a). The five choice serial reaction time task (5-CSRTT) is a behavioral task that measures the response inhibition component of executive function (5-CSRTT is also used to model aspects of attention, see below section) in mice (Robbins, 2002; Bari et al., 2008; Chudasama, 2011; Romberg et al., 2013a). The 5-CSRTT can test two different aspects of response inhibition: (1) a failure to withhold the impulsive urge to respond while anticipating correct response (premature responses) and (2) a failure to disengage from repeating past correct responses (perseveration responses) (Chudasama, 2011). Several mouse models of AD have shown deficits in response inhibition using the 5-CSRTT (Romberg et al., 2011, 2013b).

### Modeling attention

Several behavioral tasks have been developed for modeling attention in mice that provide reliable measures comparable to the neuropsychological assessments used in AD. The most widely used of these tasks is the 5-CSRTT (Muir, 1996; Humby et al., 1999; Robbins, 2002; De Bruin et al., 2006; Gibson et al., 2006; Lambourne et al., 2007; Pattij et al., 2007). This task employs an operant box with nose poke holes on the front wall of the chamber. Animals are trained to respond to brief flashes of light corresponding to five different spatial locations on this front side of the chamber and correct responses are rewarded with a food pellet released to a feeder box at the rear of the chamber. Touchscreen versions of this task are also available in which the nose poke holes and stimulus lights are replaced with an LCD screen (Romberg et al., 2011; Bussey et al., 2012). For both standard and touchscreen versions of the task, multiple trials are run each day and both the duration of the stimulus itself or the interval between the stimulus and response can be manipulated to increase the attention demands placed on the animal. Sustained attention is measured by examining when the animal responds to a different (incorrect) hole than where the stimulus light appeared (called errors of commission), fails to respond within the allotted time to the stimulus (errors of omission), and the speed with which the animal responds (reaction time). Aspects of selective attention can also be modeled with the 5-CSRTT by introducing brief bursts of white noise that the animal must ignore while still detecting the visual stimulus as it is presented (Robbins, 2002; Bari et al., 2008). The rodent 5-CSRTT is analogous to Leonard's 5-CSRTT used in humans (Wilkinson, 1963). Both tasks require subjects (mouse and human respectively) to utilize sustained attention divided among multiple spatial locations across which a large number of trials and errors of commission, omission, and reaction time are scored. Another task that can be considered somewhat analogous to the 5-CSRTT is the human Continuous Performance Tests (CPT) of sustained attention (Beck et al., 1956). In this task, the subjects are asked to respond to signal and non-signal events across numerous trials, and scores of hits, misses, rejections, and false alarms are recorded. Errors of commission in the 5-CSRTT are thought to be analogous to CPT false alarms rates. Similarly, errors of omission in the 5-CSRTT are thought to be analogous to CPT misses. AD mice such as the 3×Tg-AD mice have been shown to have deficits in sustained attention using the 5-CSRTT (Romberg et al., 2011). However, the homology between mouse and human versions of these tasks is far from perfect, and caution should be used when drawing conclusions from the rodent 5-CSRTT and applying them to human attention (Young et al., 2009).

### Modeling episodic memory

Episodic memory refers to the ability to encode and recall personal past events and experiences. Episodic memory has also been referred to as the “what, when, and where” aspect of a particular experience. Modeling AD-related deficits in episodic memory in mice is a less well-explored area than that of other aspects of working memory, executive function, or attention. Historically, episodic memory was thought to be unique to humans (Tulving and Markowitsch, 1998). However, work over the past few decades on episodic-like memory across a number of animal species has suggested otherwise, and several mouse behavioral tasks designed at assessing episodic-like memory have been developed (Clayton and Dickinson, 1998; Clayton et al., 2001; Griffiths and Clayton, 2001; Morris, 2001; Davis et al., 2013a,b). One such task is the What-Where-Which Task (WWWhich). This task is an adaptation of the NOR task. While the NOR task itself is too simplistic a task to be considered a true episodic memory task (rather it is considered a task of recognition memory), the WWWhich task is able to model episodic-like memory. In the WWWhich task, the animal must integrate the location of a particular object with specific contextual cues to form an episodic-like memory (Davis et al., 2013a,b). Several studies employing the WWWhich task have observed performance deficits related to the aging process and to AD disease state in several transgenic mouse lines (Davis et al., 2013a,b). While the WWWhich task models episodic memory in the mouse, it is not very comparable to any of the episodic memory tasks commonly used in neuropsychological testing for AD. This is largely because the human tasks rely on language as a foundational construct for assessment. Obviously, there exists no such component in the WWWhich task for mice. Therefore, caution should be used when attempting to correlate any preclinical finding concerning episodic memory in mice to that of human cognition.

### Summary of mouse behavioral tests

All of the rodent behavioral tasks discussed in this section have been specifically developed to assess deficits in cognitive domains related to what is seen in human AD. Just as multiple neuropsychological tests assessing different cognitive domains are often used clinically to provide a global cognitive profile, so multiple behavioral tasks assessing different cognitive domains should ideally be used when characterizing the profile of AD-related cognitive impairment in a particular mouse model of AD.

## Temporal progression of the cognitive deficits seen in the commonly used AD mouse models

Cognitive decline is a defining feature of AD, and many mouse models have been developed that recapitulate aspects of the cognitive impairments seen in AD (Elder et al., 2010; Hochgrafe et al., 2013; Platt et al., 2013). Although no one animal model fully replicates the progression of cognitive impairments seen in the human disease, AD mouse models have been invaluable in advancing our knowledge of the disease. It should be noted that most of the AD mouse models are representative of the familial form of AD (FAD) which accounts for only a small percentage of the total AD cases each year (Campion et al., 1999). In addition, the contributions of both background strain and likely overexpression of mutant human APP genes on brain development and function must always be considered with regard to observed cognitive deficits in the various AD mouse models. Each transgenic mouse model of AD provides different insights into aspects of AD pathogenesis and the cognitive deficits associated with the disease. A generalized time course of the development of cognitive deficits across the various mouse models is depicted in Figure 1B. For each specific mouse model the temporal time course and progression of cognitive deficits in each cognitive domain can be different. In addition, in some models, cognitive deficits can be detected prior to the appearance of significant neuropathology. Careful forethought is therefore required in the selection of an optimal model displaying the AD related cognitive deficits desired based on the specific research interests of the investigator. An overview of the progressive cognitive deficits and the time of appearance of amyloid pathology is presented in Table 3 for five mouse models that contain amyloid precursor protein (APP) mutations and in Table 4 for five other common mouse models that contain APP and presenilin (PS1) mutations, or APP/PS1/Tau mutations. These tables are by no means an all-encompassing list of mouse models; rather they are simply meant to be examples of some of the commonly used mouse models of AD that are characterized by APP mutations. For recent reviews of additional AD mouse strains not included here, see (Ashe and Zahs, 2010; Elder et al., 2010; Epis et al., 2010; Hall and Roberson, 2012; Platt et al., 2013).

**Table 3:**
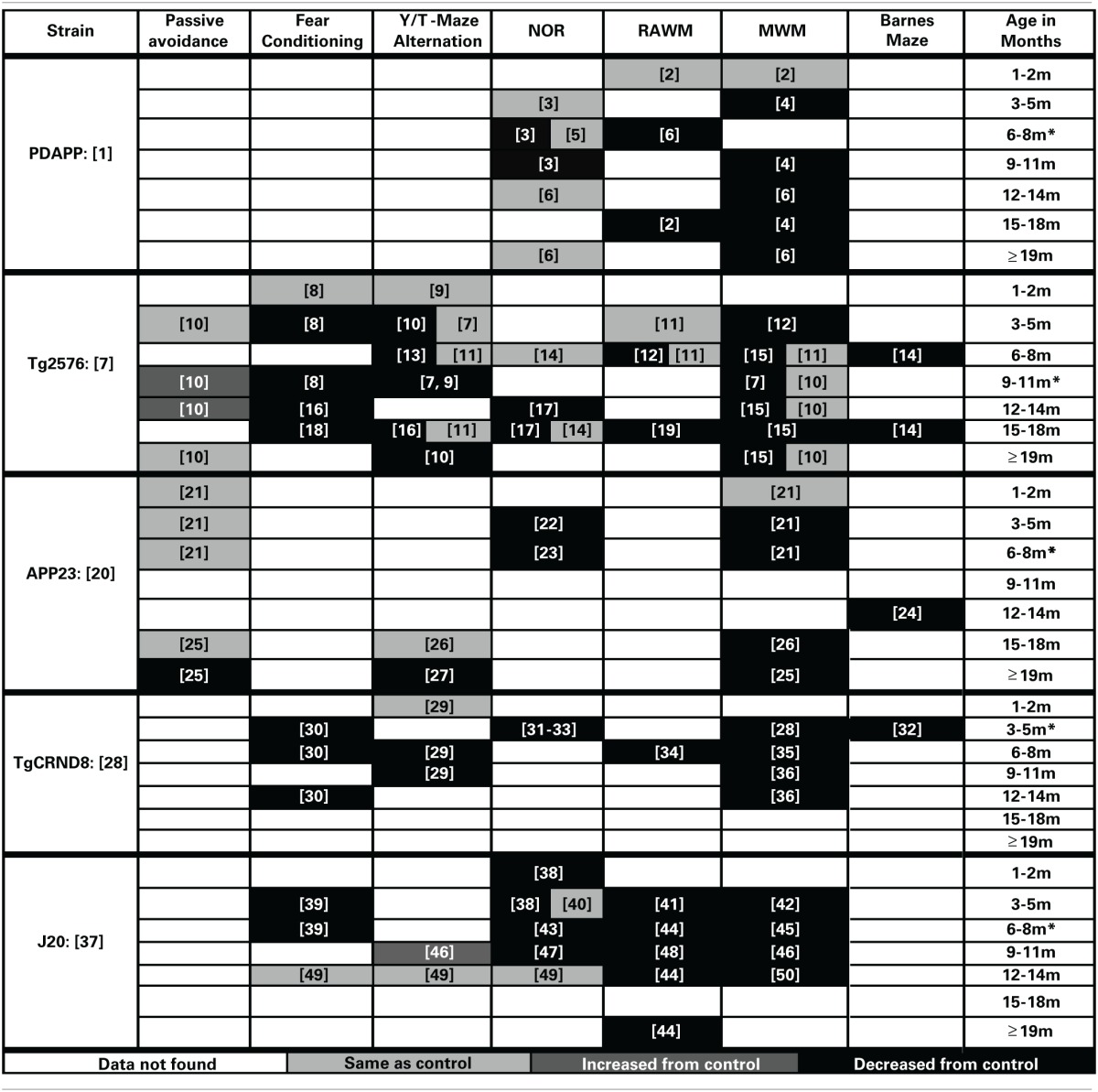
**Progression of cognitive deficits in APP mouse models of AD**.

The strain is presented in the left column and age is presented in the right column. Black cells represent impairment, light gray cells represent no impairment compared to controls, dark gray cells represent increases from control (very few such cases), and white cells represent time points where no data are available for the respective behavior. The asterisk appearing in the age column represents when diffuse amyloid plaques are first observable in the brain for that particular mouse model of AD. The numbers within each cell correspond to the following references: 1: (Games et al., 1995), 2: (Nilsson et al., 2004), 3: (Dodart et al., 1999), 4: (Hartman et al., 2005), 5: (Dodart et al., 2002), 6: (Chen et al., 2000), 7: (Hsiao et al., 1996), 8: (Dineley et al., 2002), 9: (Chapman et al., 1999), 10: (King et al., 1999), 11: (Arendash et al., 2001a), 12: (Arendash et al., 2004), 13: (Ohno et al., 2004), 14: (Yassine et al., 2013), 15: (Westerman et al., 2002), 16: (Corcoran et al., 2002), 17: (Oules et al., 2012), 18: (Lassalle et al., 2008), 19: (Morgan et al., 2000), 20: (Sturchler-Pierrat et al., 1997), 21: (Van Dam et al., 2003), 22: (Huang et al., 2006), 23: (Heneka et al., 2006), 24: (Prut et al., 2007), 25: (Kelly et al., 2003), 26: (Lalonde et al., 2002), 27: (Dumont et al., 2004), 28: (Chishti et al., 2001), 29: (Hyde et al., 2005), 30: (Hanna et al., 2012), 31:(Ambree et al., 2009), 32: (Gortz et al., 2008), 33:(Richter et al., 2008), 34: (Lovasic et al., 2005), 35: (Janus, 2004), 36: (Hanna et al., 2009), 37: (Mucke et al., 2000), 38: (Harris et al., 2010), 39: (Saura et al., 2005), 40: (Simon et al., 2009), 41: (Lustbader et al., 2004), 42: (Cheng et al., 2007), 43: (Cisse et al., 2011), 44: (Du et al., 2011), 45: (Palop et al., 2003), 46: (Murakami et al., 2011), 47: (Escribano et al., 2009), 48: (Fang et al., 2012), 49: (Karl et al., 2012), 50: (Galvan et al., 2006).

**Table 4:**
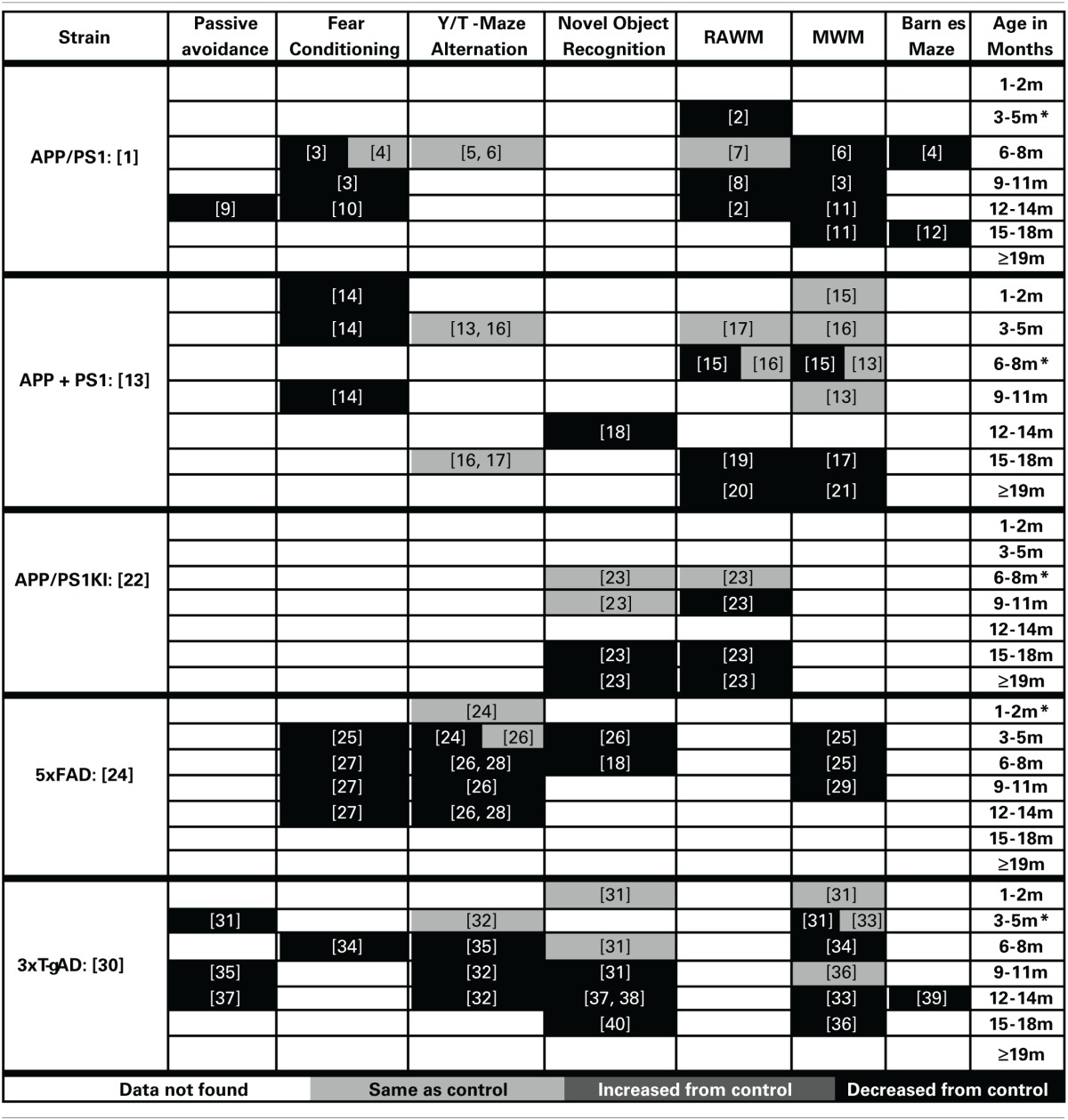
**Progression of memory deficits in other mouse models of AD (APP + PS1/Tau)**.

The strain is presented in the left column and age is presented in the right column. Black cells represent impairment, light gray cells represent no impairment compared to controls, dark gray cells represent increases from control (very few such cases), and white cells represent time points where no data are available for the respective behavior. The asterisk appearing in the age column represents when diffuse amyloid plaques are first observable in the brain for that particular mouse model of AD. The numbers within each cell correspond to the following references: 1: (Jankowsky et al., 2001), 2: (Park et al., 2006), 3: (Cramer et al., 2012), 4: (Reiserer et al., 2007), 5:(Cao et al., 2007) 6:(Lalonde et al., 2004), 7: (Volianskis et al., 2010), 8: (Sood et al., 2007), 9: (Zhang et al., 2011), 10: (Knafo et al., 2009), 11: (Lalonde et al., 2005), 12: (O'leary and Brown, 2009), 13: (Holcomb et al., 1999), 14: (Dineley et al., 2002), 15: (Trinchese et al., 2004), 16: (Arendash et al., 2001a), 17: (Arendash et al., 2001b), 18: (Tohda et al., 2012), 19: (Morgan et al., 2000), 20: (Wilcock et al., 2004), 21: (Sadowski et al., 2004), 22: (Flood et al., 2002), 23: (Webster et al., 2013), 24: (Oakley et al., 2006), 25: (Ohno et al., 2006), 26: (Shukla et al., 2013), 27: (Devi and Ohno, 2010), 28:(Devi and Ohno, 2012), 29: (Urano and Tohda, 2010), 30: (Oddo et al., 2003), 31: (Clinton et al., 2007), 32: (Carroll et al., 2007), 33: (Gimenez-Llort et al., 2007), 34: (Billings et al., 2005), 35: (Nelson et al., 2007b), 36: (Pietropaolo et al., 2008), 37: (Filali et al., 2012), 38: (Arsenault et al., 2011), 39: (Stewart et al., 2011), 40: (Halagappa et al., 2007).

### PDAPP

(Promoter: Platelet-Derived (PDGF) Promoter, Symbol: Tg (APPV717F) 109Ili, MGI ID: 2151935)

The PDAPP mouse was first described by Games in 1995 and is considered one of the earliest mouse models of AD (Chartier-Harlin et al., 1991; Games et al., 1995). In this model, the cognitive deficits first present themselves in spatial working memory at 4 months of age when assessed by MWM testing (Hartman et al., 2005). These deficits in working memory in the MWM are present throughout the rest of the life span for this model (Chen et al., 2000; Daumas et al., 2008). Deficits in recognition memory appear to develop after the working memory deficits in this model, as the first reported deficits in the NOR task are at 6 months old (Dodart et al., 1999). The cognitive deficits in recognition memory do not appear as robust as those in working memory in this model as there are inconsistent reports in the literature (Dodart et al., 1999, 2002; Chen et al., 2000). The cognitive defects in this model appear to present themselves before the appearance of plaque deposition (first appear at approximately 6 months) or other gross amyloid pathologies (Games et al., 1995; Hsiao et al., 1996; Schenk et al., 1999; Chen et al., 2000; Morgan, 2003).

### TG2576

(Promoter: Hamster PrP Promoter, Symbol: Tg (APPSWE) 2576Kha, MGI ID: 2385631)

In the Tg2576 mouse (Hsiao et al., 1996), the first presentation of cognitive deficits is seen at 5 months of age in spatial working memory (Arendash et al., 2004). These spatial working memory deficits are generally accepted to be present across the rest of the life span for this model (Hsiao et al., 1996; Westerman et al., 2002; Arendash et al., 2004). However, methodology used to assess spatial memory appears to be very important, as several different reports have failed to observe these same deficits at various ages (Hsiao et al., 1996; Arendash et al., 2001a; King and Arendash, 2002). Non-spatial working memory tasks show a similar time course progression, first appearing at 3–5 months of age and persisting across the lifespan (Hsiao et al., 1996; Chapman et al., 1999; King and Arendash, 2002; Lalonde et al., 2003; Ohno et al., 2004). Deficits in recognition memory do not appear until much later, first appearing at 12 months of age in the NOR task (Oules et al., 2012; Yassine et al., 2013).

### APP23

(Promoter: Thy-1, Symbol: Tg (Thy1-APP) 3Somm, MGI ID: 2447146)

The APP23 mouse model was reported in 1997 (Sturchler-Pierrat et al., 1997). In this model, the cognitive deficits begin to first appear in both recognition memory and spatial working memory at 3 months of age. The deficits appear to be progressive with age, and at 12 months old the animals also show cognitive deficits in a reference memory version of the Barnes maze (Prut et al., 2007). This model develops non-spatial working memory deficits very late in the progression of the disease (only after 19 months of age) (Lalonde et al., 2002; Dumont et al., 2004). Interestingly, cognitive performance in passive avoidance memory tasks follows the same progression as non-spatial working memory deficits in this model, unimpaired at 15 months of age and then developing deficits between 19 and 20 months of age (Kelly et al., 2003).

### TgCRND8

(Promoter: PrP, Symbol: Tg (PRNP-APPSweInd) 8Dwst, MGI ID: 3589475)

The TgCRND8 model, described by Chishti et al. (2001), exhibits early cognitive impairment that spans across multiple cognitive domains (Chishti et al., 2001). TgCRND8 are impaired on spatial working memory tasks starting at 3 months of age. These deficits are seen in the MWM and progress with age of the animal (Janus et al., 2000; Chishti et al., 2001; Gortz et al., 2008; Richter et al., 2008; Ambree et al., 2009). Reference memory deficits via Barnes maze testing are also present at 3 months of age (Gortz et al., 2008; Richter et al., 2008; Ambree et al., 2009). Similar to this observed temporal time course of spatial working memory and reference memory deficits are the development of deficits in both recognition memory and fear conditioning (Gortz et al., 2008; Richter et al., 2008; Ambree et al., 2009; Hanna et al., 2012). Deficits in alternation tasks develop by 6 and 9 months in Y-maze and T-maze alternation tasks, respectively (Hyde et al., 2005).

### J20

(Promoter: Platelet-Derived (PDGF), Symbol: Tg (PDGFB-APPSwInd) 20Lms, MGI ID: 3057148)

The J20 mouse model was developed by (Mucke et al., 2000). This model is unique in that the first presented cognitive deficits are observed very early (at 1–2 months of age) in recognition memory (Harris et al., 2010). These deficits in recognition memory are present when assessed at several other time points (Escribano et al., 2009; Simon et al., 2009; Cisse et al., 2011). However, they do not appear to progress with the age of the animal and there has even been a report of no recognition memory deficits in old animals in advanced stages of the disease (Karl et al., 2012). Early memory deficits can also be observed in spatial working memory at 3 months of age when assessed by the MWM and the RAWM tasks (Lustbader et al., 2004; Cheng et al., 2007; Meilandt et al., 2008). These spatial working memory deficits are present across the rest of this model's lifespan (Palop et al., 2003; Galvan et al., 2006; Cisse et al., 2011; Du et al., 2011; Murakami et al., 2011; Fang et al., 2012). Fear conditioning deficits appear consistent with the presentation of spatial working memory and recognition memory deficits in this model (Saura et al., 2005). Interestingly, this model does not appear to display working memory deficits on tasks of alternation such as the Y-maze (Murakami et al., 2011; Karl et al., 2012).

### APP/PS1

(Promoter: PrP, Symbol: Tg (APPswe, PSEN1dE9) 85Dbo, MGI ID: 3524957)

The cognitive deficits in the APP/PS1 mouse model, first described by Jankowsky et al. (2001), have been well characterized. Cognitive deficits are first seen at 3 months of age in the RAWM spatial working memory task and are also reported by 6 months of age in the MWM (Cao et al., 2007; Ding et al., 2008). Further, these deficits have been well characterized across the lifespan of this mouse model in water based spatial working memory tasks (Lalonde et al., 2005; Park et al., 2006; Cao et al., 2007; Sood et al., 2007; Ding et al., 2008; Volianskis et al., 2010; Zhang et al., 2011; Cramer et al., 2012; Ma et al., 2012). Impairments in reference memory develop by 6 months and persist through the rest of the life of this model (Reiserer et al., 2007; Bernardo et al., 2009; O'leary and Brown, 2009). Deficits in associative learning have also been described in fear conditioning tasks starting at 6–8 months of age (Knafo et al., 2009; Cramer et al., 2012). Similarly, passive avoidance deficits have also been described at 12 months of age (Zhang et al., 2011). No deficits were seen in alternation tasks of working memory for this model (Lalonde et al., 2004; Cao et al., 2007).

### APP + PS1

(Promoter: Hamster PrP Promoter, Symbol: Tg (APPSWE) 2576Kha, MGI ID: 2385631) × (Platelet-Derived (PDGF), Symbol: Tg (PDGFB-PSEN1M146L) 2Jhd, MGI ID: 2447326)

Holcomb described a mouse model in 1998 that has been widely used to study cognitive deficits related to AD (Holcomb et al., 1998). The first observable deficits in this model are shown in associative learning and present themselves between 4 and 5 months of age (Dineley et al., 2002). The progression of the spatial working memory impairment in this model is relatively slow compared to most other models. The first reported impairment in spatial working memory was observed using 6-month-old animals (Trinchese et al., 2004). However, these cognitive deficits are not robust at this age, as others have observed no such deficit (Holcomb et al., 1999; Arendash et al., 2001a). By 15 months of age the spatial working memory is consistently impaired throughout the rest of the life span (Morgan et al., 2000; Arendash et al., 2001b; Gordon et al., 2001; Sadowski et al., 2004; Wilcock et al., 2004). Similarly, deficits in recognition memory occur later in this model, first observed at 12 months of age (Mori et al., 2013). No deficits were observed in alternation tasks of working memory (Holcomb et al., 1998; Arendash et al., 2001a).

### APP/PS1 KI

(Promoter: Endogenous, Symbol: Apptm1.1Cep, MGI ID: 2652346) × (Promoter: Endogenous, Symbol: Psen1tm1Dgf, MGI ID: 3608968)

The APP/PS1 knock-in mouse model (first described in Flood et al., 2002) uses endogenous promoters to drive the expression of humanized amyloid beta sequence, and AD-like pathology and cognitive deficits develop in the absence of APP or PS1 overexpression (Flood et al., 2002). The earliest reports of cognitive deficits are reported at 7 months in this model (Bruce-Keller et al., 2011). However, the majority of cognitive deficits appear later. We have shown previously that the cognitive deficits in spatial working memory (assessed by RAWM testing) first appear at 9 months of age (Webster et al., 2013). These deficits are followed by impairments in associative memory (appearing by 14 months of age Thibault et al., 2012) and in recognition memory (not developing until 15 months of age Webster et al., 2013).

### 5×FAD

(Promoter: Thy-1, Symbol: Tg (APPSwFlLon, PSEN1^*^M146L^*^L286V) 6799Vas, MGI ID: 3693208)

The 5×FAD model, first described by Oakley et al. (2006), develops progressive cognitive deficits with age. This model develops cognitive deficits by 3 months of age in spatial working memory (Ohno et al., 2006; Urano and Tohda, 2010). These working memory deficits are followed temporally with the development of associative learning impairment in fear conditioning (Ohno et al., 2006; Devi and Ohno, 2010) as well as the development of deficits in a working memory version of the Y-maze (Oakley et al., 2006; Devi and Ohno, 2012; Shukla et al., 2013). As with several of the other models (PDAPP, Tg2576, APP/PS1, and APP/PS1 KI) this model develops deficits in recognition memory later than the observed deficits in spatial working memory (Tohda et al., 2012).

### 3×Tg-AD

(Promoter: Thy-1, Symbol: Tg (APPSwe,tauP301L) 1Lfa, MGI ID: 2672831) × (Promoter: Endogenous, Symbol: Psen1tm1Mpm, MGI ID: 1930937)

The 3×Tg-AD mouse model, developed by Oddo et al. (2003), shows progressive cognitive impairments starting at a young age. The first deficits observed in this model are those of associative learning deficits, which begin between 3 and 5 months of age. These are then followed by deficits in spatial working memory at 6 months of age in the MWM task. Both Y-maze alternation and contextual fear conditioning impairment follow a similar temporal time course. Then deficits in recognition memory present themselves between 9 and 11 months of age. Finally, reference memory impairment in the Barnes maze task is observed at 12 months of age.

## Temporal progression of AD-like non-cognitive behavioral abnormalities seen in the commonly used mouse models

While most AD research has focused on the neurobiological mechanisms underlying the cognitive deficits seen in AD pathogenesis, there is a wide range of non-cognitive neuropsychiatric symptoms also associated with the disease. Indeed, these non-cognitive symptoms are seen as a very important concern among the family members of the patients and caregivers alike (Tan et al., 2005). These non-cognitive symptoms are often more difficult to deal with, as they compose important sources of distress and psychological burden on the family members/caregivers and can drastically affect the quality of life of patients by leading to institutionalization (Hope et al., 1998; Shin et al., 2005). Non-cognitive neuropsychological symptoms associated with AD include activity disturbances, affective disturbances, aggression, stereotypic behavior, circadian rhythm disturbances, and anxiety (Ancoli-Israel et al., 1989; Okawa et al., 1991; Vitiello et al., 1992; Bliwise, 1994; Satlin et al., 1995; Van Someren et al., 1996; Hope et al., 1998; Harper et al., 2004; Shin et al., 2005; Tan et al., 2005). While there has been less emphasis placed on the modeling of these non-cognitive neuropsychological disturbances in murine models of AD, several of the commonly used mouse models of AD do show a number of these disruptions. Non-cognitive symptoms associated with AD shown in the mouse include increased locomotor activity (Dodart et al., 1999; King et al., 1999; Arendash et al., 2001b; Dumont et al., 2004; Hyde et al., 2005; Cheng et al., 2007; Gil-Bea et al., 2007; Pietropaolo et al., 2008; Sanchez-Mejia et al., 2008; Ambree et al., 2009; Cisse et al., 2011; Mori et al., 2013), anxiety (Moechars et al., 1996, 1999; Lalonde et al., 2003, 2004; Gil-Bea et al., 2007; Reiserer et al., 2007; Lassalle et al., 2008; Espana et al., 2010; Bedrosian et al., 2011; Cisse et al., 2011; Murakami et al., 2011; Filali et al., 2012), circadian disturbances (Huitron-Resendiz et al., 2002; Vloeberghs et al., 2004; Wisor et al., 2005; Ambree et al., 2006; Sterniczuk et al., 2010; Bedrosian et al., 2011), and increased aggression (Moechars et al., 1996, 1998; Van Dorpe et al., 2000; Ambree et al., 2006; Vloeberghs et al., 2006; Pugh et al., 2007; Alexander et al., 2011).

### Locomotor activity

Most of the commonly used AD mouse models exhibit increased locomotor activity (Dodart et al., 1999; King et al., 1999; Arendash et al., 2001b; Dumont et al., 2004; Hyde et al., 2005; Cheng et al., 2007; Gil-Bea et al., 2007; Pietropaolo et al., 2008; Sanchez-Mejia et al., 2008; Ambree et al., 2009; Cisse et al., 2011; Mori et al., 2013). These disturbances include hyperactivity, stereotypic behaviors, and home cage activity disturbances (Dodart et al., 1999; Janus and Westaway, 2001; Auld et al., 2002; Dumont et al., 2004; Hyde et al., 2005; Cheng et al., 2007; Gil-Bea et al., 2007; Gimenez-Llort et al., 2007; Pietropaolo et al., 2008; Cisse et al., 2011) and have been linked to altered APP metabolism, amyloid levels, and disease progression (Van Someren et al., 1996; Harper et al., 2004). These activity disturbances do not seem to be constant, but rather present themselves with more severity at different times of the day. For example, in the TgCRND8 mice, the deficits seem most severe near the end of their wake cycle and less severe at other times of the day (Ambree et al., 2006). Further, these disturbances increase with the age of the animals and with the severity of the disease. Likewise, in the APP23 mice there have been reports of increased activity in the second half of the nocturnal phase (end of the active phase of the wake cycle) (Vloeberghs et al., 2004). These increased activity disturbances at the end of the activity cycle have been suggested to be similar to the exacerbation of activity behavioral symptoms observed in human AD patients late in the afternoon and evening time termed sundowning syndrome (Vitiello et al., 1992; Bliwise, 1994). Other AD mouse models that show activity disturbances are the PDAPP, TG2576, J20, APP + PS1 [Tg2576 + PS1 (M146L)], and 3×Tg-AD mouse models of AD. The onset of the disturbances is different for each model, with the J20 and the TgCRND8 mice developing disruptions earliest (approximately 1 month of age), followed by PDAPP and TG2576 (approximately 3 months of age), and the PS1 [Tg2576 + PS1 (M146L)], 3×TgAD, and APP23 mice developing last (approximately 6–9 months of age) (Dodart et al., 1999; King et al., 1999; Arendash et al., 2001b; Dumont et al., 2004; Hyde et al., 2005; Cheng et al., 2007; Gil-Bea et al., 2007; Gimenez-Llort et al., 2007; Pietropaolo et al., 2008; Sanchez-Mejia et al., 2008; Ambree et al., 2009; Harris et al., 2010; Cisse et al., 2011; Mori et al., 2013).

### Circadian rhythm and sleep disruptions

Similar to the reported activity disturbances, circadian rhythm disruptions are also observed in many of the AD mouse models (Huitron-Resendiz et al., 2002; Vloeberghs et al., 2004; Wisor et al., 2005; Ambree et al., 2006; Sterniczuk et al., 2010; Bedrosian et al., 2011). Circadian rhythm disturbances have also been well described in human AD patients (Ancoli-Israel et al., 1989; Okawa et al., 1991; Vitiello et al., 1992; Van Someren et al., 1996; Auld et al., 2002; Harper et al., 2004). These disturbances are characterized by the AD patient's propensity to frequently awaken during the nighttime and to increase the amount of time slept during the day (Ancoli-Israel et al., 1989; Okawa et al., 1991). While this behavior is in itself a non-cognitive behavior, it has been reported to possibly have important effects on patient cognition (Smith, 1985; Graves et al., 2001). Several mouse models of AD also display sleep disruption. Other similarities between the sleep disturbances seen clinically and those seen in the AD mouse models are: (1) as the severity of the disease progresses, the worse the sleep disturbances become (Smith, 1985; Graves et al., 2001; Huitron-Resendiz et al., 2002, 2005) and (2) the greater the sleep disruptions are, the more severe the cognitive decline seems to be (Huitron-Resendiz et al., 2002, 2005).

### Anxiety disturbances

Anxiety disturbances have been reported in many of the AD mouse models (Moechars et al., 1996, 1999; Lalonde et al., 2003, 2004; Gil-Bea et al., 2007; Reiserer et al., 2007; Lassalle et al., 2008; Espana et al., 2010; Bedrosian et al., 2011; Cisse et al., 2011; Murakami et al., 2011; Filali et al., 2012). The prevailing thought is that these anxiolytic-like behaviors stem from disinhibitory tendencies resulting from the underlying AD pathology (Lalonde et al., 2003; Ognibene et al., 2005). Both APP function (as these anxiolytic behaviors are more common in APP transgenic mice) (Moechars et al., 1996, 1999; Lalonde et al., 2003, 2004; Lassalle et al., 2008; Murakami et al., 2011; Filali et al., 2012) and disruption of the cholinergic system (Apelt et al., 2002; Klingner et al., 2003; Luth et al., 2003) because of its well-known role in behavioral inhibition (and disruption in several of the AD mouse lines) have been proposed as underlying causes of this behavioral abnormality. The temporal time course of these anxiety-like disturbances can vary depending on the mouse model. For example, in some models the behavioral disturbances start early at 1–2 months of age or 3–6 months of age for the J20 and the APP/PS1 models, respectively (Lalonde et al., 2004; Reiserer et al., 2007; Harris et al., 2010). Other models such as the TG2576 develop the anxiety-like disturbances later at the age of 9–11 months of age (Gil-Bea et al., 2007). It is also important to note that not all murine AD models exhibit anxiety disturbances (Arendash et al., 2001b; Lalonde et al., 2002; Webster et al., 2013). Still, the majority of models do display these disturbances and the prevailing thought is that this behavioral phenotype of disinhibition may be akin to the disinhibition seen in AD patients (exemplified by unacceptable behavior and inappropriate euphoria) (Daffner et al., 1992; Chung and Cummings, 2000).

### Aggressive behaviors

Increased aggressive behaviors are another common behavioral symptom of AD and present themselves in as much as 65% of AD patients (Burns et al., 1990b). As with other non-cognitive behavioral symptoms of AD, increased aggression can be emotionally stressful to both the patient and caregivers (Murman et al., 2002a,b). The exact mechanism that underlies these increased aggressive behaviors is not known but proposed mechanisms deal with dysregulation of different neurotransmitter systems such as serotonin, norepinephrine, dopamine, and GABA (Arsland, 1995; Meltzer et al., 1998). Many AD mouse models also display increased aggressive behavior (Moechars et al., 1996, 1998; Van Dorpe et al., 2000; Ambree et al., 2006; Vloeberghs et al., 2006; Pugh et al., 2007; Alexander et al., 2011). For example, in the TG2576 mouse increased aggressive behaviors display themselves in both the frequency of attacks on other home cage mice as well as on the latency to first attack when interacting with a novel mouse (Alexander et al., 2011). The APP23 mouse model of AD also shows increased aggressive behaviors (Vloeberghs et al., 2006). These aggressive disruptions appear to develop later than the onset of cognitive deficits in this model (Kelly et al., 2003; Van Dam et al., 2003; Vloeberghs et al., 2006). The aggression alterations in the APP23 model appear by 6 months of age (after amyloid pathology and behavioral deficits) and seem to remain relatively constant throughout the rest of the course of disease (Vloeberghs et al., 2006). This suggests that perhaps aggressive deficits correlate best with moderate to severe stages of the disease, which is also what is observed clinically in human AD (Senanarong et al., 2004).

### Depressive symptoms

Depressive symptoms/behaviors are a very common comorbidity with AD. The exact prevalence of this comorbidity is not known but is believed to range from as low as 2% to as high as 85% (variability likely due to methods of assessment, diagnostic criteria, stage of AD participants, and other factors) (Mendez et al., 1990; Burns et al., 1990a; Migliorelli et al., 1995a,b; Devanand et al., 1996; Cummings, 2000; Apostolova and Cummings, 2008). Numerous meta-analysis studies have linked depression and AD (Chen et al., 1999; Charlson and Peterson, 2002; Ownby et al., 2006; Lenoir et al., 2011; Diniz et al., 2013), and several even consider late-life depression a significant risk factor for future development of AD (Butters et al., 2000, 2008; Diniz et al., 2013). Generally, depressive symptoms precede the onset of AD (Devanand et al., 1996) and usually worsen with the progression of the disease (Mega et al., 1996). Similarly, patients with a history of depression prior to a diagnosis of AD are much more likely to experience depressive episodes in the course of AD (Pearlson et al., 1990; Strauss and Ogrocki, 1996). Despite this well documented connection between depression and AD and having a wide range of potentially applicable tools to study depression in animal models (Seligman et al., 1975; Jolly et al., 1999; Song and Leonard, 2005; Flint and Shifman, 2008; Nestler and Hyman, 2010), very little work has been devoted to determining the range of depressive behavioral symptoms in the commonly used mouse models of AD. Depressive-like behaviors have been reported in at least one mouse model of AD (Filali et al., 2009), and likely exist in many of the other commonly used models.

## Conclusions

No one animal model fully replicates the pathogenesis of AD, but rather only model different aspects of the disease. Consequently, no one model recapitulates all of the cognitive deficits observed in human AD. Further, the anatomical makeup and cognitive ability of mice make it difficult to model all of the intricacies of higher-order cognitive function exclusive to humans. Instead, each mouse model allows us insight into different aspects of cognition related to AD. Several important points should be taken away from the preceding discussion of the temporal development of cognitive deficits in the various mouse models of AD. First, the temporal time course and progression of cognitive deficits in a specific cognitive domain/behavioral task can be quite different among the different mouse models. Investigators should use careful forethought in selection of an optimal model and planning experiments based on the progression of that model's specific deficits. Secondly, most models display deficits in spatial working memory earlier than the deficits in other cognitive domains. Similarly, most of the studies using mouse models of AD have focused on understanding/correcting the cognitive deficits associated with the disease. However, AD is not just a memory disorder, rather it is a complex disease with many different non-cognitive neuropsychiatric symptoms which are an important source of distress and a psychological burden on family members and caregivers alike. These non-cognitive symptoms are present across many of the different mouse models of AD and more emphasis should be placed on understanding/correcting these deficits, as well as the cognitive aspects of the disease. It is our hope that this comprehensive review of the spectrum of behavioral deficits present in commonly used AD mouse models and how well they model human cognitive and non-cognitive symptoms will assist investigators in selecting an appropriate mouse model to investigate different aspects of AD pathology and disease progression.

### Conflict of interest statement

The authors declare that the research was conducted in the absence of any commercial or financial relationships that could be construed as a potential conflict of interest.
